# Benefits of Using Design Patterns on Microcontrollers in Implemented IoT Applications

**DOI:** 10.3390/s24237803

**Published:** 2024-12-05

**Authors:** Marek Babiuch, Petr Foltynek

**Affiliations:** Department of Control Systems and Instrumentation, VSB—Technical University of Ostrava, 70800 Ostrava, Czech Republic; petr.foltynek.st@vsb.cz

**Keywords:** design pattern, ESP32, framework, IoT, microcontroller, programming, sensor, SOLID

## Abstract

As part of our research for microcontroller software support, we have developed a modular framework that utilizes previously unimplemented architectural principles for developing applications on microcontrollers. These principles are still a privilege of enterprise and server applications. The paper describes the benefits of a new architectural approach to developing applications on microcontrollers and describes the most common application scenarios along with examples of IoT application development using a framework with design pattern architecture and SOLID principles. As a result, our framework supports developers in creating robust, adaptive, and scalable applications. It emphasizes a modular and clean design that increases development efficiency and enables easy deployment of new features or integration of new technologies, such as new types of sensors, upgraded development boards, or improved development tools and frameworks. The architectural concepts offered useful guidance for creating applications ready for future challenges and changing technology environments, especially in the IoT area.

## 1. Introduction

Historically, procedural programming was the first and most widespread approach to programming microcontrollers because they were originally designed for simple tasks such as controlling sensors, motors, and other peripherals. Although advances in hardware architecture are enormous, many developers working with microcontrollers still prefer this procedural approach despite its current and increasing shortcomings.

**Figure 1:**
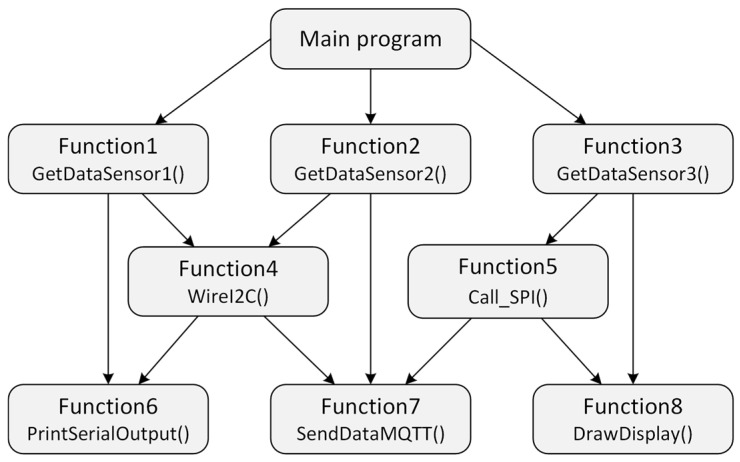
Structure of the procedure-oriented program.

Among the main disadvantages of procedural programming on microcontrollers are poor modularity, limited portability of code and its almost impossible reusability, complexity of memory management, low level of abstraction, poor scalability, and, last, but not least, difficult testing and debugging. The structure of bindings in a procedure-oriented program, calls, and communication between functions is shown in Figure 1.

A program designed in this way is difficult to maintain. Each modification of the program increases its complexity. The program is gradually reaching a state where the costs of adding new functions will increase so much that it is no longer worth expanding the program.

There is an intermediate stage between procedural and object-oriented programming called modular programming. Modular programming allows us to encapsulate certain functionality into modules. Although it brings us a better application structure, more is needed to eliminate the disadvantages of procedural programming.

**Figure 2:**
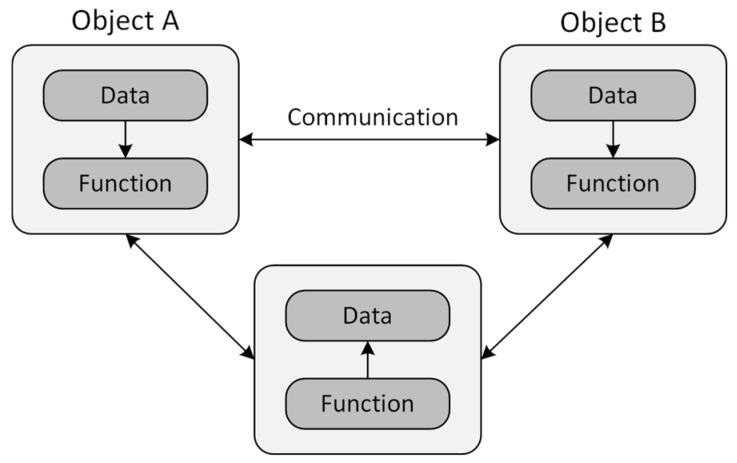
Organization of data and functions in an object-oriented approach.

Overall, procedural programming on microcontrollers requires careful attention to detail and knowledge of hardware aspects. This can be very challenging, so the alternative to using an object-oriented approach is often offered to address some of the shortcomings of procedural programming. An object-oriented approach brings several significant advantages, particularly code reuse, an efficient and transparent concept, and flexibility in creating more complex data structures because of inheritance and polymorphism [1]. Figure 2 shows the arrangement of data and functions in object-oriented programs.

Programs implemented using object-oriented principles satisfy the following properties:

- The emphasis is on data rather than procedures.
- Programs are divided into so-called objects.
- Data structures are designed to characterize objects.
- Functions that work with object data are bound to the data structure.
- The object itself controls the accessibility of data in an object.
- The data are hidden and external functions can only access the data if the object allows it.
- Objects can communicate with each other through functions and well-defined interfaces.
- New data and functions can easily be added through the inheritance mechanism.
- However, this mechanism can be influenced by declaring a class.
- A bottom-up approach to program design is applied.

To understand object-oriented programming, it is necessary to understand the following concepts: Objects, Classes, Data Abstraction and Encapsulation, Inheritance, Polymorphism, Dynamic Binding, and Message Passing [2].

An object is a fundamental building element in an object-oriented system. Objects can represent, in our case, a sensor, a location, measured data, or any entity that the program works with. Objects can also represent user-defined data such as vectors, time, or lists of elements. The program design is analyzed in terms of the objects and the nature of communication between them. The objects of the program should be chosen to closely match the requirements for real-world objects. When the program starts, objects communicate by sending messages to each other. Each object contains data and code for manipulating the data. Objects can communicate with each other without needing to know the details of their data or code. You just need to know the type of message and the type of response the objects return.

A class is the basic building block of object-oriented programming. The class serves as a prescription for creating an object through an instance of the class. Once a class is defined, we can create any number of objects belonging to that class. The class defines the data (attributes) and functions (methods) of the objects created when the program runs. The values of properties (attributes) can differ for individual object instances, so we can call them the same set of functions on each object. Encapsulation is the wrapping of data and functions into a single entity called a class. The primary purpose of a class is to encapsulate data and functions. This implies that the data are inaccessible from the outside world, and, thus, the data can only be accessed through functions that are part of the class. These functions provide an interface between the object data and the program. The data isolation from direct program access allows creating an impenetrable wall that protects the code against unwanted changes.

Abstraction is a way of generalizing some details of the work of individual objects. Each object works like a black box that can perform specified actions and interact with its environment without requiring knowledge of its internal workings. Classes using the concept of abstraction are defined as a list of abstract attributes and functions that work with those attributes. They encapsulate all the essential properties of the object to be created.

**Figure 3:**
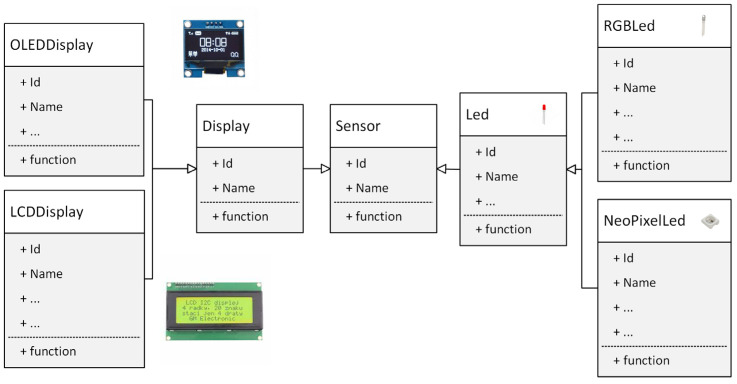
Using inheritance in sensor modules.

Inheritance is the process by which objects of one class acquire the properties of objects of another class. It supports the concept of hierarchical classification. Let us have a general sensor class that describes the given sensor using its name and unique identification (e.g., sensor serial number). Then, the “RGBLed” class is part of the “Led” class, which is derived from the “Sensor” class (see Figure 3). The principle of this kind of division is that each derived class shares common properties with the class from which it is inherited. In the object-oriented approach, the concept of inheritance provides the idea of reusability. That is, we can add additional properties to an existing class without having to modify it. This is possible by deriving a new class from an existing class.

**Figure 4:**
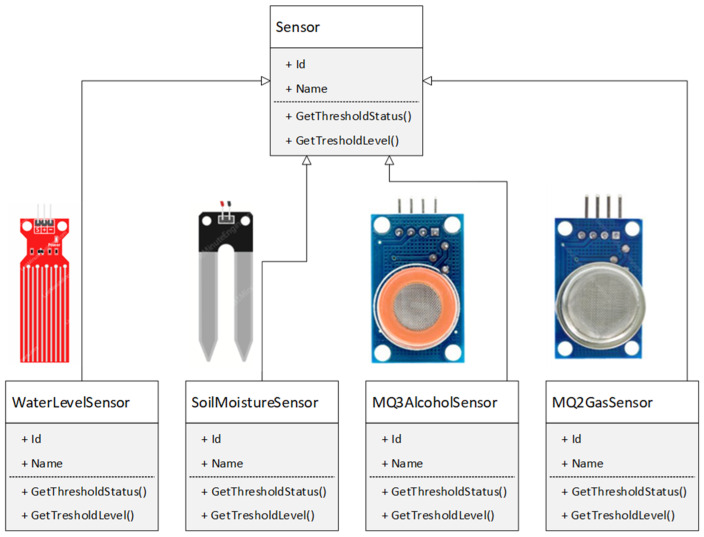
Polymorphic behavior of derived sensor classes from the Sensor base class.

Another important concept of the object-oriented approach is polymorphism. Polymorphism is a Greek term meaning the ability to take on more than one form. A function can exhibit different behavior in different instances. The behavior of a function depends on the type of data used in the function. Polymorphic behavior is implemented in different programming languages at a different level of abstraction, fulfilling the basic idea of overriding a function in a child derived from a base class [3]. The process by which a function exhibits different behaviors in different cases is known as function override. Thus, in the context of C/C++, overriding is a technique that enables polymorphism, and polymorphism is a concept that uses overriding to achieve flexible and extensible code. For example, consider the “GetTresholdStatus()” function, which displays a sensor’s status after it has exceeded a certain level related to a specific physical quantity. Figure 4 illustrates that one function name can be used to operate different types of sensors.

Polymorphism plays an important role in allowing objects that have different internal structures to share the same external interface. Polymorphism is widely used in the implementation of inheritance.

Dynamic binding refers to linking a function call to the code to be executed in response to the call. It means that the code associated with a given function call is not known until the call is made at runtime. Dynamic binding is associated with polymorphism and inheritance. Calling a function associated with a polymorphic reference depends on the dynamic type of that reference. Consider, for example, the “GetThresholdLevel()” function shown in Figure 4. Due to inheritance, this function will be implemented in every object, but its algorithm is unique for each object. Hence, the “GetThresholdLevel()” function is defined in every class that defines an object. At runtime, the code corresponding to the object instance will be called.

**Figure 5:**
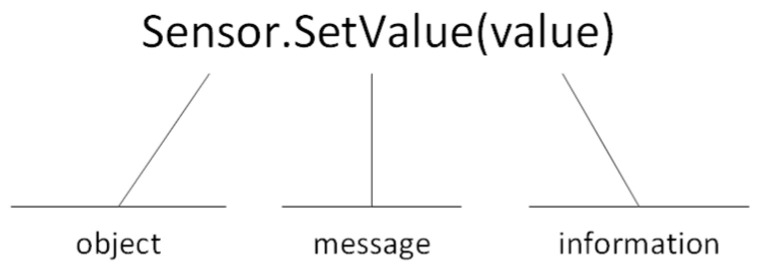
Passing messages in the object.

Objects communicate with each other by sending and receiving information in the same way that people send messages. A message to an object is a request to perform a function. To send a message, you need to enter the object name, function name, and information parameter (see Figure 5). Since an object has its own life cycle, it can be created and destroyed, so communication with the object is only possible while the object is alive.

**Figure 6:**
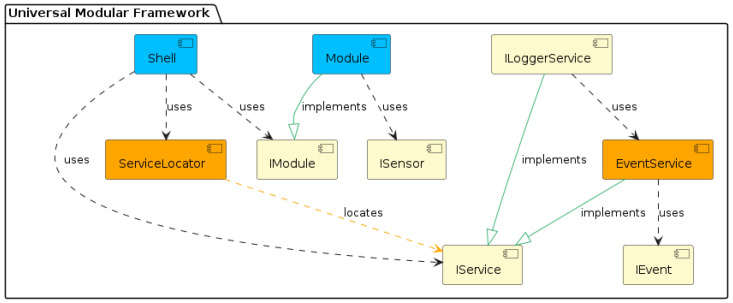
Universal Modular Framework for Microcontrollers.

The object-oriented approach offers several advantages for the program designer and the user. Object orientation contributes to solving many problems related to the development and quality of software products. This approach promises greater programmer productivity, better software quality, and lower maintenance costs. Together with the power of the object-oriented approach, it is desirable to design applications for microcontrollers using SOLID [4,5] principles, which serve as a guide for solving different program design variants. In developing our universal framework for microcontrollers, we went even further and decided to also apply design patterns [6,7]. Implementation using design patterns helps developers achieve better code quality, more manageable code maintenance, and increased code reusability, which, in our case, we consider to be a huge benefit and a technique not yet applied in the field of developing applications for microcontrollers and embedded systems [8]. This universal modular framework, which uses SOLID principles and design patterns, is shown in the component diagram in Figure 6.

A very detailed description of the implementation of this universal modular framework, created using SOLID principles and design patterns using the C++ language, is described in the article [9]. The modularity of the framework represents a clearly defined structure of modules. It separates application logic from the physical implementation of sensors, describes a mechanism for interaction between loosely coupled modules, and, among other things, introduces the Sensor–View design pattern we created, supporting platform independence.

The framework is divided into separate functional modules implemented through the *Module* component. These modules are independent and can freely interact with each other via the *EventService* component. Each communication event uses *IEvent* components. The modules contain some of the application’s functionality, such as application logic, sensor communication, or infrastructure services. We use our proposed *Sensor–View* design pattern [9] to provide abstract access to the sensors, which implements the *ISensor* component. The *Shell* component is designed to secure the registration of independent modules within a single application. The *ServiceLocator* component is used here to implement the DI (*Dependency Injection*) container [10]. This approach facilitates the application’s development, testing, deployment, and maintenance because each module can be developed and tested independently while contributing to the system’s overall functionality. In the article [9], we presented its concrete, detailed implementation, including the responsibility of individual components. We recommend getting to know this framework’s implementation, which is presented and includes sample implementations of individual components at the source code level. In the following sections of this article, we will show the benefits of using this framework in various specific application scenarios.

## 2. Using Modular Framework in Microcontroller Applications

This section will focus on the practical use of our modular framework in microcontroller applications.

Projects are in various environments and with different hardware requirements. We present a scenario where a developer needs to implement the same functionality, for example, collecting and processing data from a sensor providing environmental measurements from the following microcontrollers: ESP32 [11], ESP8266 [12], RPI–Pico [13], and STM32 [14].

In our second example, we will then explore how our modular framework provides platform independence. We demonstrate this on the following two popular development platforms: Arduino and the Espressif IoT Development Framework (ESP–IDF) from Espressif Systems. Testing will take place on a circuit similar to the one used in the first scenario, except that we will choose an ESP32 as the microcontroller for testing here. This comparison will help us better understand how our framework enables consistent and efficient development across different platforms.

In our third example, we will focus on the resilience of a modular framework to changes in hardware module types while keeping the application logic consistent. We present a scenario where the circuitry is similar to the previous cases, but the hardware modules for data visualization and measurement of environmental variables are replaced. We show how the framework allows easy replacement of one hardware module with another without requiring significant changes in the application layer. This flexibility is practically usable when developing different versions of the same device that differ in detection accuracy. This allows developers to respond efficiently and flexibly to market demands or technological advances without redesigning the entire application.

Our last example demonstrates a reference implementation of our modular framework within a complex application. This project will illustrate how different hardware modules can be effectively integrated into a unified system, where each module is implemented as a separate and replaceable unit. This approach ensures a high level of flexibility and extensibility of the application. Each module is designed to function independently and easily integrate into the overall system without the need for significant code changes. This allows developers to add new features or improve existing ones without the need for a complete redesign of the entire application. Such a framework becomes an invaluable tool for developers looking for a flexible and sustainable solution for their IoT and home automation projects.

### 2.1. Validation of the Framework Deployment on Different Types of Development Boards

**Figure 7:**
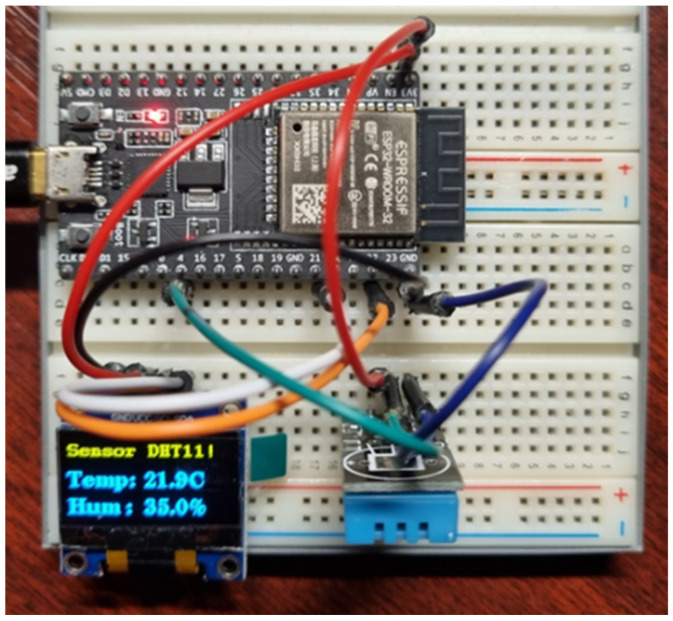
Wiring the task on the ESP32-WROOM-32 microcontroller.

In the first example, we will show how our modular framework handles running on different types of microcontrollers. This behavior will be demonstrated using an example where the microcontroller collects data on temperature and air humidity using a DHT11 sensor and subsequently visualizes these data using an OLED display SSD1306 with a resolution of 128 × 64 pixels. We chose this example because of the popularity of the hardware used in IoT projects in home automation, weather stations, and other applications requiring essential temperature and humidity data. The DHT11 sensor is a digital device capable of measuring temperature between 0 °C and 50 °C with an accuracy of ±2% and relative humidity between 20% and 90% with a tolerance of ±5%. This sensor usually has the following three pins for connection: VCC (power), typically in the range of 3.3 to 5 V; data (signal): for data transfer between the sensor and the micro-controller using a 1-Wire bus; and a GND pin. This three-pin layout makes the DHT11 sensor easy to integrate with different types of microcontrollers. Another hardware module used in our example is the OLED 0.96” SSD1306 display with a resolution of 128 × 64 pixels, as shown in Figure 7. It is a high-contrast, organic LED display designed for sharp and clear display. Communication with the microcontroller is in progress via the I2C interface. It is a popular serial communication protocol requiring only two pins—SCL (Clock Line) and SDA (Data Line). Thanks to the I2C interface, this display can be easily integrated into various microcontroller-based projects, offering developers flexibility and simplicity when designing user interfaces. This OLED display is ideal for projects where compactness, low power consumption, and high visual quality are essential.

This section focuses on implementing an application that uses our proposed modular framework with the modules described above. The first microcontroller on which we will demonstrate this implementation is the Espressif ESP32-WROOM-32. The connection of this microcontroller is shown in Figure 7.

**Figure 8:**
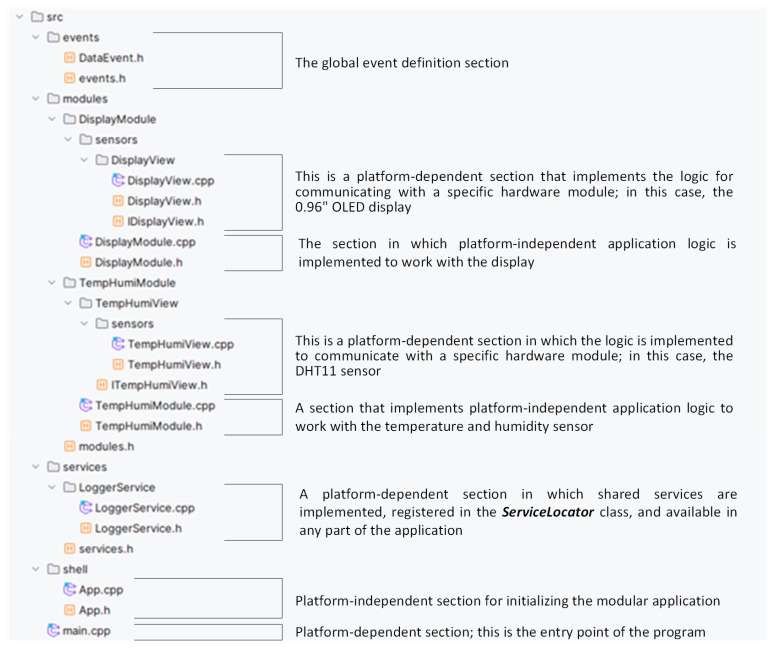
Modular structure of the program.

The main goal is to create an application that efficiently reads data from the DHT11 sensor and then visualizes these data on a connected display, allowing users to view current environmental conditions easily. To achieve this goal, we follow a recommended application structure that is designed to make the most of the principles of modularity and code clarity. The structure of the project, illustrating the layout of the single-individual modules, is shown in Figure 8.

As you can see in Figure 8, the program is divided into two modules that logically represent hardware components. The first module is responsible for reading data from the temperature and humidity sensor, and the second module is responsible for displaying these values on the display. To ensure independent communication between modules, it is mediated using the *EventService* class, where one module provides an event and the other subscribes to it. We use the Sensor–View design pattern to ensure that the application is independent of specific hardware and that each module can operate independently. This pattern allows us to separate the application logic from the hardware specifics. We now look at implementing the module that handles temperature and humidity information acquisition.

**Figure 9:**
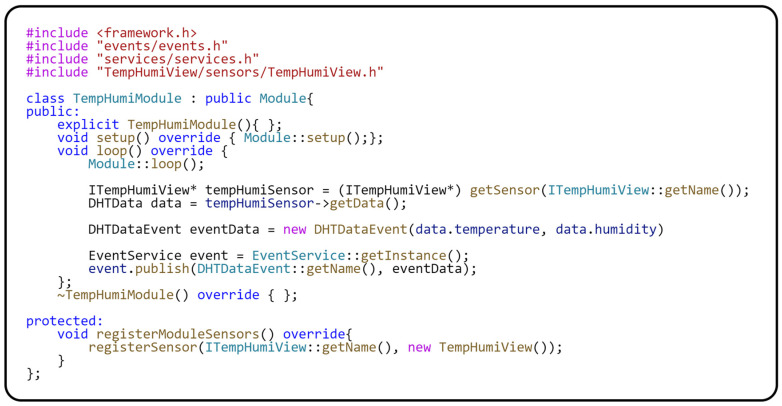
Implementation of the module for measuring environmental values.

Looking at the attached code in Figure 9, there is no direct interaction with the platform-dependent sensor within the implemented module. The class for communicating with the sensor is registered in the *registerModuleSensors()* method. This method of integration demonstrates critical aspects of modular architecture, such as dependency separation and a high degree of context separation. An important role within the module is played by the *loop()* method, which represents the central execution loop. During this loop, an abstraction is used to get data from the sensor, specifically through the *getSensor(…)* method. This method ensures obtaining an instance of the abstract interface of the sensor, which allows the separation of the application logic from the specifics of the given hardware.

**Figure 10:**
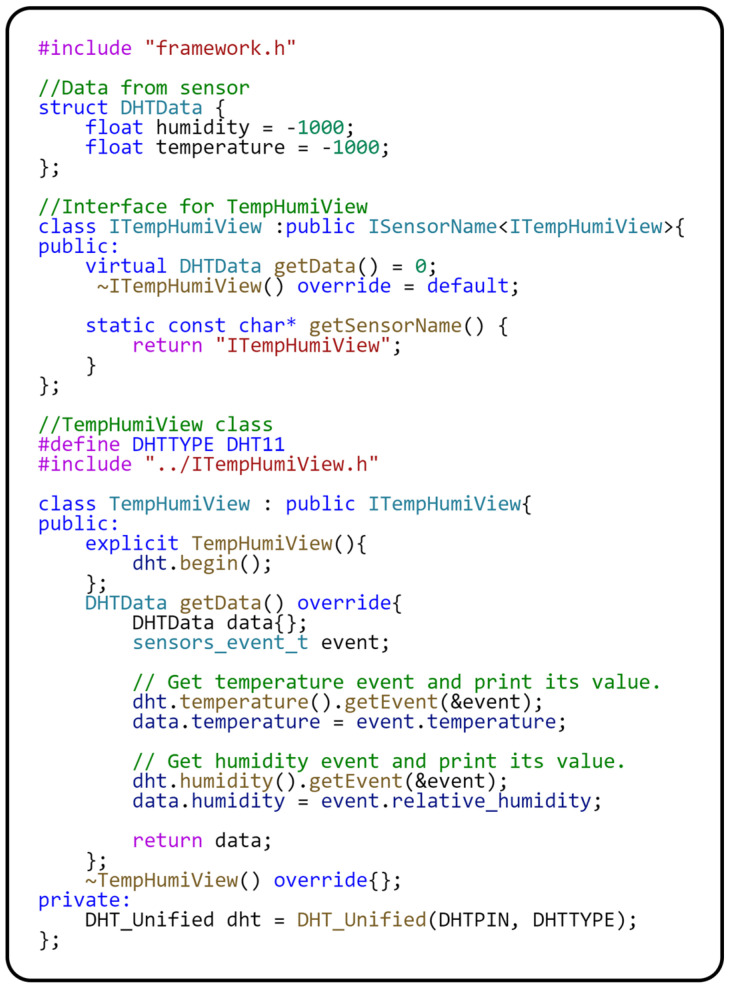
Implementation of the Sensor–View design pattern for the DHT11 sensor.

The *getData()* function of the mentioned interface provides measured values in the form of an internal structure, which minimizes dependencies and increases the modular nature of the application. The publication of the obtained data for the entire application is realized through the global event *DHTDataEvent*. This mechanism enables other modules to react to the actual data. In the last part of the *loop()*, this event is published using the EventService class, which involves transforming the data from a local data structure into a structure suitable for global sharing and processing. The implementation also includes a *LoggerService* for logging key information during operation. This service is essential for monitoring and debugging the application, highlighting the importance of your role in maintaining the application. The next step is to analyze the implementation of the Sensor–View design pattern for the DHT11 physical sensor (see Figure 10).

**Figure 11:**
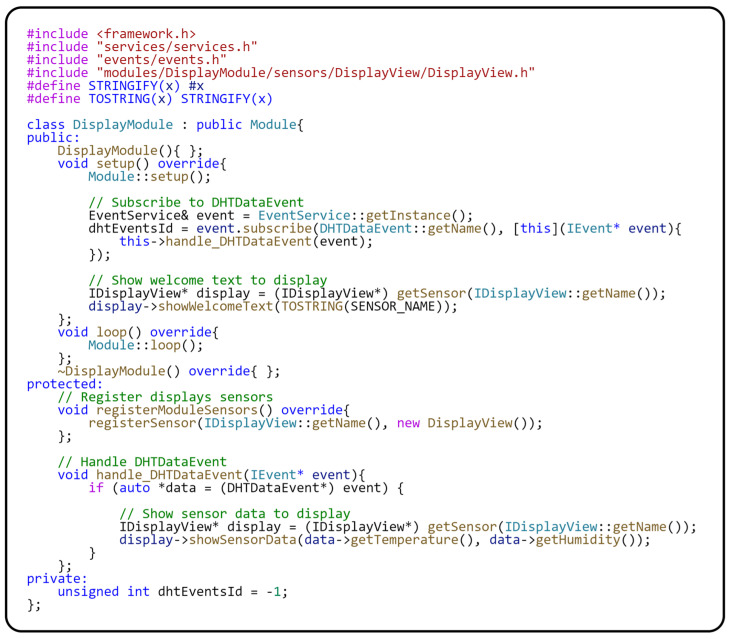
Implementation of a module for displaying measured values.

The Sensor–View design pattern [9] is used for communication with the sensor to implement the independence of the application logic stored in the module. The primary role in implementing the Sensor–View design pattern is represented by the *ITempHumiView* interface, which contains the *getData()* method for obtaining data from the sensor. Specifically, the *getData()* method of the *TempHumiView* class interacts directly with the DHT11 sensor. This interaction is implemented through the standard sensor driver so that the *getData()* method gets the current temperature and humidity data directly from the sensor. It is important to note that the initialization of the DHT11 sensor is performed in the constructor of the *TempHumiView* class using the *dht.begin()* statement. For the correct operation of the driver, it is necessary to define which GPIO pin the sensor is connected to, which is performed through the DHTPIN macro. This greatly improves clarity and makes the code easier to maintain since the physical sensor connection is defined centrally and, thus, not spread throughout the program. Now that we have described the module responsible for data acquisition, let us focus on the *DisplayModule*, responsible for displaying data on the screen. The implementation of this module in the source code is provided in Figure 11.

**Figure 12:**
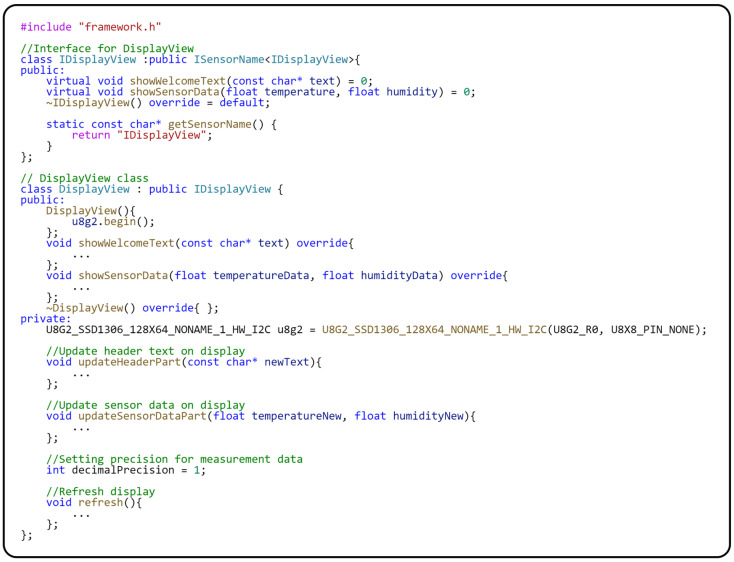
Implementation of the Sensor–View design pattern for OLED display.

The principles used in this module are similar to those implemented in the previously described module. The *registerModuleSensors()* method registers the class to communicate with the sensor. We will now take a closer look at the *setup()* method, which is used to initialize the module. The first part of the *setup()* method subscribes to the global *DHTDataEvent* provided by the *EventService* class. Here, you can see that subscribing to this event activates the *handle*_*DHTDataEvent(…)* method, in which the data from the argument of the given method is obtained. The data are then displayed on the display using the Sensor–View design pattern. The *showSensorData(…)* method displays the data to which the measured parameters are passed. The *handle_DHTDataEvent(…)* method is called whenever a new data set is published via the *publish()* method in the *TempHumiModule*. In the second part of the setup method, access to the sensor (display) is obtained through the *getSensor(…)* method. The *showWelcomeText()* method is then called on this sensor, which displays the name of the sensor type providing the measured data. The sensor type is specified using the SENSOR_NAME macro. The implementation of the *Sensor–View* pattern for communicating with the display is in the following source code in Figure 12.

The interface for communication between the application logic represented by the model and the hardware element (display) implements the following two methods: *showWelcomeText(…)* and *showSensorData(…)*. We have previously described the behavior of both methods. The *DisplayView* class implements communication with the display through a standard driver for displays.

**Figure 13:**
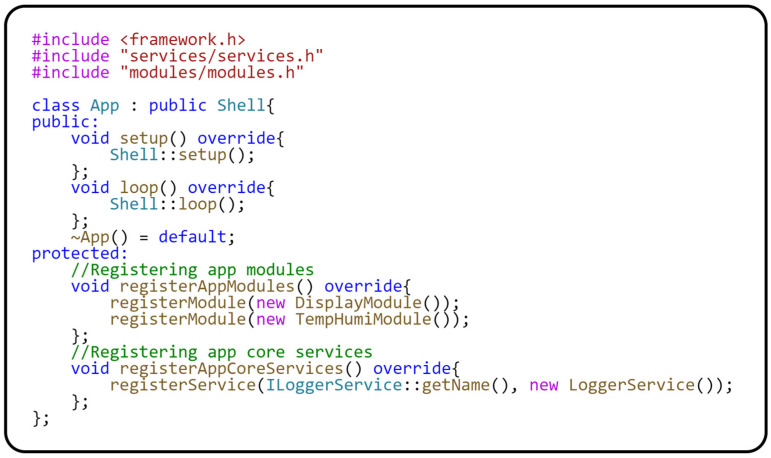
Registration of the module and core services into the application context.

Now that we have described the behavior of the modules and their hardware dependencies, we can move on to the description of the *App* class. This class is inherited from the *Shell* class and implemented within the Modular framework. The *Shell* class implements general module handling. The *App* class implementation is platform-independent and fully shielded from any hardware dependencies. The following code in Figure 13 shows the implementation of the *App* class.

The implementation of the *App* class is relatively simple. The most interesting parts are found in the *registerAppCoreServices()* method, which registers generic services that are used throughout the application. Another important method is *registerAppModules()*, which registers the individual modules of the application. It is important to note that the order in which modules are registered plays a key role, as the *setup()* and *loop()* methods of each module are called in that particular order.

**Figure 14:**
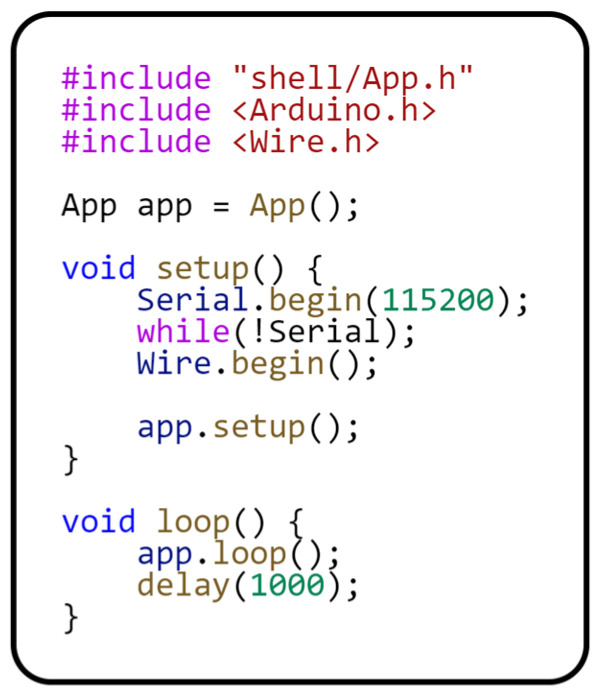
Application entry point.

The last piece of code we will introduce is the application entry point. This entry point, which is highly platform-dependent, ensures the initialization and launch of the entire application. The following code in Figure 14 demonstrates this entry point.

**Figure 15:**
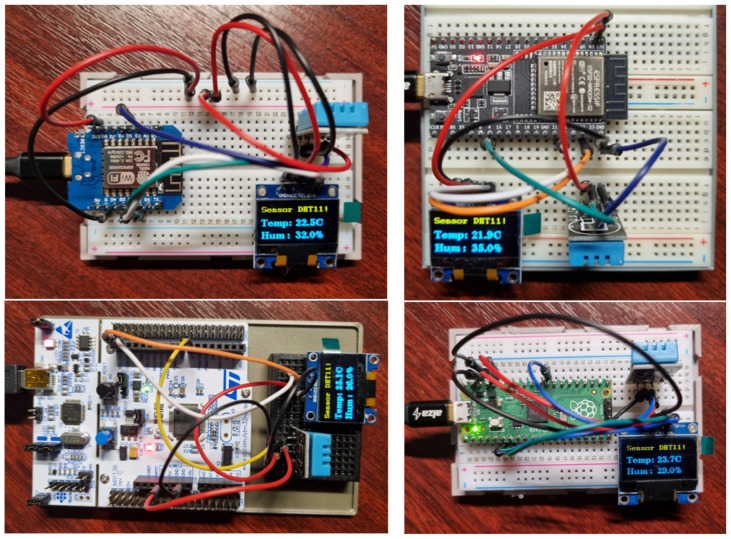
Application deployed on different types of microcontrollers.

The *setup()* method initializes the serial link communication and the I2C protocol. The *loop()* method is repeated every 1 s, corresponding to the measurement period. We subsequently deployed the same project on the following microcontrollers shown in Figure 15. Since we are using different microcontrollers, each circuit is slightly different. Mainly, it is the connection of the DHT11 sensor data pin.

**Figure 16:**
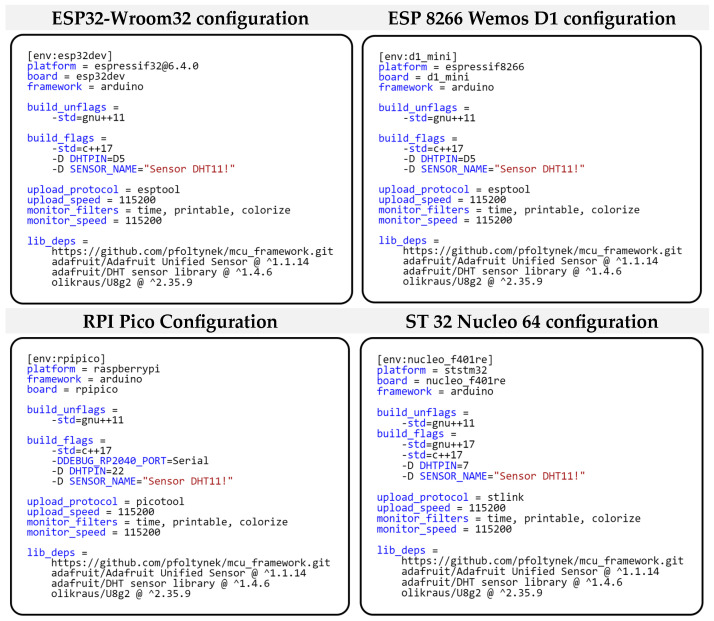
Project configuration for different types of microcontrollers.

We solved this variable setting through the preprocessor directive switch “-D,” which is used when compiling the program. Thus, the main change in deploying our framework on different microcontrollers is only in the configuration of the given project. Figure 16 shows the project configurations for each microcontroller. As you can see from the individual configurations, the configurations differ only in the DHTPIN switch. We can also notice that all projects are set to support C++ 17. This setting is required by our modular framework, which is built on modern features and paradigms of this standard, such as advanced template functions, lambda-da expressions, and strong-type safety. These allow for the creation of a more efficient and readable code. In the project configuration, we further define dependent libraries that provide not only drivers for hardware peripherals, but also the framework we developed. We currently provide the library for the framework developed by us using a link to the GitHub project [15]. We then use a standard public repository to obtain the remaining libraries. We use the “U8g2” library to communicate with the OLED display. For communication with the DHT11 sensor, we use the “DHT sensor library,” which requires the “Adafruit Unified Sensor” library for its correct functionality. This library provides a unified interface for working with different types of sensors.

**Figure 17:**
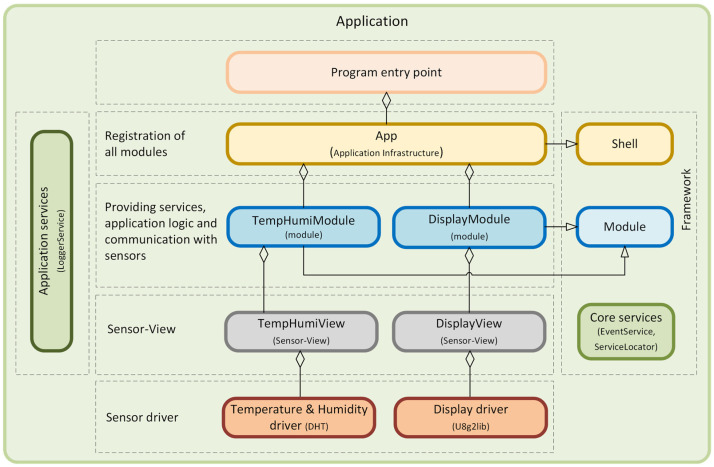
Component Responsibility Diagram.

This test case was specifically designed to showcase the adaptability of our framework to various microcontrollers. Figure 17 visually represents the described example. It shows the dependencies of the individual components in the program that were used in the source code for this example. The different layers of responsibility of the application are also clearly visible. The results unequivocally demonstrate that our framework can be seamlessly deployed on different platforms, providing us with a wide range of options. Whether it is replacing one microcontroller with another or transitioning from one generation of devices to another, this adaptability is a cornerstone of our long-term sustainable projects. It empowers us to swiftly respond to evolving requirements and technological advancements, making it an indispensable feature for efficient and sustainable application development in the dynamic IoT environment. The complete source code for this example is available on the GitHub portal [16].

### 2.2. Verification of Platform Independence of the Framework on Different Types of Development Platforms (Arduino vs. ESP–IDF)

In the first example, we demonstrated how to use our modular framework on different types of microcontrollers. We now focus on showing how our framework can be effectively used to develop applications on various development platforms. The example on which we will perform the demonstration is the same as in the previous section. Within this example, we will focus primarily on the effects that the implementation of this example has within the framework of different development platforms. We chose the following two development platforms for comparison: Arduino and Espressif IoT Development Framework (ESP–IDF). To ensure consistency, both implementations use the same type of microcontroller, “ESP32-WROOM-32,” using the identical hardware components detailed in the previous example. Thus, the project’s structure remains the same as in the previous example in Figure 8.

**Figure 18:**
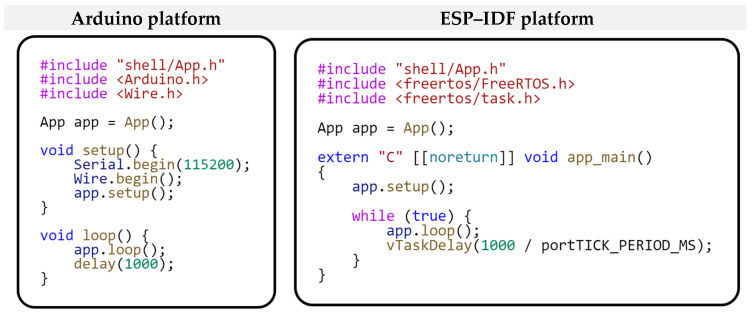
Application entry point deployed on different development platforms.

Our focus in this comparison will be on the platform-dependent part. This is because the application logic implemented within the modules remains unchanged from the previous example, allowing us to focus directly on the differences arising from these development platforms’ specific characteristics. We will start comparing individual platforms from the application’s entry point. Figure 18 shows how application entry points differ for different development platforms. We already described the entry point for the Arduino platform in the previous example. The application entry point for the ESP–IDF framework uses a more standard programming approach. At the beginning of the *app_main* method, the *setup()* method of the *App* class is called, where the whole modular framework is initialized. This is followed by creating an infinite loop using *while(true)*, in which the *loop()* method of the App class is called periodically. The last statement within this loop is a call to the *vTaskDelay(…)* function, which will pause the loop for a certain amount of time. In our case, this is to pause the program for one second. The *vTaskDelay(…)* function corresponds to the *delay()* function from the Arduino platform. As you can see, even though the entry points of the applications differ in their notation, their meaning and functionality are the same. The application entry point is defined as platform-dependent within the project structure. However, this platform dependency does not affect the application logic that is implemented within the modules.

**Figure 19:**
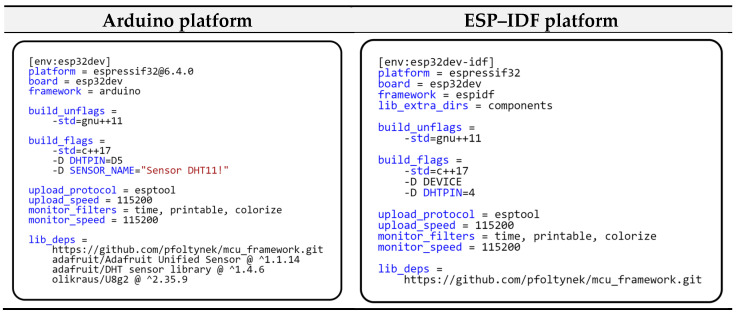
Project configuration for different platforms.

Looking at the configuration of individual projects, we immediately notice several differences. Figure 19 shows the project configuration for each platform. In the previous example, we have already explained the configuration items for the Arduino platform; now, we will focus on the specifics of the configuration for the ESP–IDF framework. The main configuration difference for ESP–IDF is that it uses only one library, our modular framework. Unlike the Arduino development platform, ESP–IDF does not use built-in libraries for hardware peripheral drivers by default. To ensure communication with these hardware components, it is necessary to implement the appropriate components that solve this approach.

**Figure 20:**
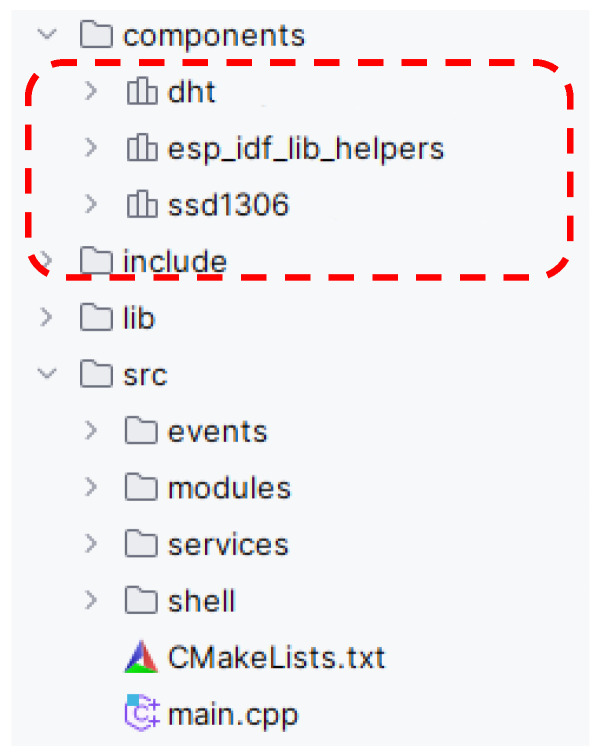
The only difference in the project structure on the ESP–IDF platform.

Therefore, we added these components to our project and defined where this component directory is located within the configuration. This configuration corresponds to the entry “*lib_extra_dirs = components*”. Figure 20 shows the structure of the ESP–IDF project. The only change from the previous example is the addition of the “components” directory, which defines the drivers to access the DHT11 temperature and humidity sensor and to communicate with the OLED 0.96” display.

Since the ESP–IDF framework uses different drivers for communication with hardware peripherals than the Arduino platform, modifying other platform-dependent parts of the code is necessary. First, we will focus on the module that handles the temperature and humidity sensor. To communicate with this sensor, we need to reimplement the *TempHumiView* class. This class represents the sensor part of the Sensor–View design pattern implemented within the *TempHumiModule*.

The second platform-dependent part that needs to be modified is the *DisplayView* class. This class represents the sensor part in the Sensor–View design pattern and is used within the *DisplayModule*. The module is responsible for rendering the values obtained from the DHT11 sensor to the display.

**Figure 21:**
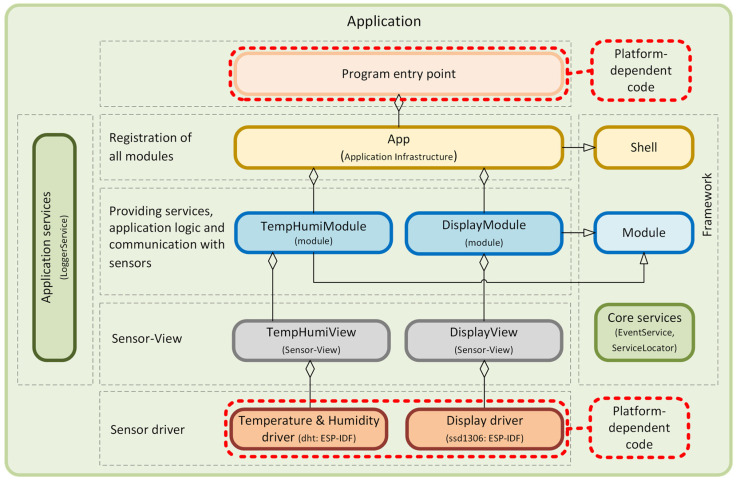
Component responsibility diagram after platform change.

Due to the use of different drivers for our hardware peripherals, the visual appearance of the data displayed on the display is slightly different. It is clear from this comparison that when changing the development platform, it is necessary to modify only the platform-dependent parts of the program. The application logic implemented within the modules remains unchanged. Figure 21 shows the visual representation of these changes. The complete source code for the entire application is available on the project’s GitHub portal [16].

This example confirms that our modular framework is applicable across different development platforms, which underlines the versatility and flexibility of the approach we use in our framework. Thus, the principles applied within our modular framework are highly generic and platform-independent, allowing for their wide application in diverse development contexts. This ability is critical to an efficient and flexible application development process.

### 2.3. Verification of the Universality of the Framework When Sensor Modules Are Changed

**Figure 22:**
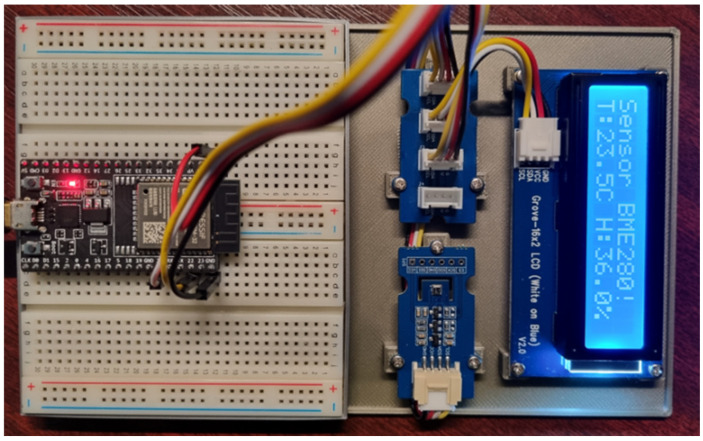
Wiring a task on the ESP32-WROOM-32 microcontroller with Grove modules.

In the first example, we demonstrated the ability of our modular framework to work on different types of microcontrollers, while in the second example, we demonstrated its compatibility with different development platforms. We now focus on demonstrating the robustness of our framework to changes in the types of hardware peripherals. For this purpose, we will use the same circuitry as in the first example but with a change of hardware peripherals. Here, we use the Grove–BME280 sensor to measure temperature and humidity. The Grove–BME280 sensor is a powerful multifunctional module that measures temperature, humidity, and atmospheric pressure. Based on Bosch’s accurate and efficient BME280 chip, this module offers high measurement accuracy with tolerances of ±1 °C for temperature, ±3% RH for humidity, and ±1 hPa for atmospheric pressure. In addition, it features low power consumption. This sensor uses an I2C interface to communicate with the microcontroller, which enables simple and efficient integration into various projects. To display the measurement results, we use the Grove-16 × 2 LCD module. The Grove-16x2 LCD module uses an I2C interface to communicate with the microcontroller, allowing easy wiring and programming. This display is suitable for a wide range of applications. We are now focusing on implementing an application that implements our modular framework along with the above modules. The microcontroller we will use for this example is the Espressif ESP32-WROOM-32. The circuit implementation of this microcontroller is shown in Figure 22.

In this example, we use the same Espressif ESP32-WROOM-32 microcontroller as in our first example, but only the hardware peripherals have been changed. As a result of this change, we will focus primarily on the platform-dependent part of our application. Crucially, the application logic remains identical to the one we used in the first example. This approach allows us to examine in more detail how our framework reacts to changes in hardware components while maintaining consistency in the application logic. The functionality and structure of the application remain the same. In this way, we can effectively demonstrate how our application adapts to hardware changes without having to modify its basic logic.

**Figure 23:**
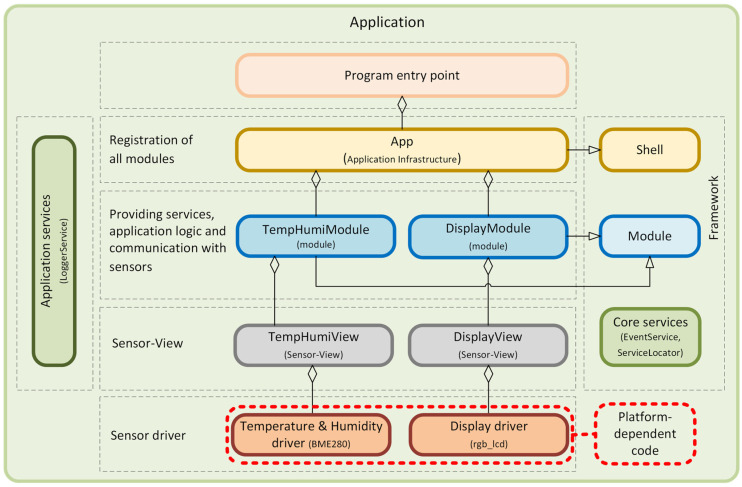
Diagram of component responsibility when changing sensors.

The first step in adapting our application to the new hardware configuration is the reimplementation of the platform-dependent part, which is represented by the *TempHumiView* class. This class represents the sensor part of the Sensor–View design pattern. As can be seen from the responsibility diagram in Figure 23, the BME280 sensor is used to obtain data on air temperature and humidity. Although this sensor measures atmospheric pressure, our application does not currently use this value. If we wanted to integrate support for measuring atmospheric pressure into the application, it would be necessary to make corresponding adjustments in the application logic to process this value efficiently. Another platform-dependent part we need to re-implement is the *DisplayView* class, which is responsible for displaying data on the display. This class is part of the Sensor–View design pattern and integrated into the *DisplayModule*. As can be seen in Figure 23, the *DisplayView* class uses drivers for a 16 × 2 LCD. Since this display offers a smaller display space than a 0.96” OLED display, this aspect must be considered when reimplementing the class. These readings are abbreviated to display the temperature and humidity readings effectively, and the first line of the display shows the name of the sensor used to measure the temperature and humidity. Figure 22 then clearly shows how these values are displayed.

**Figure 24:**
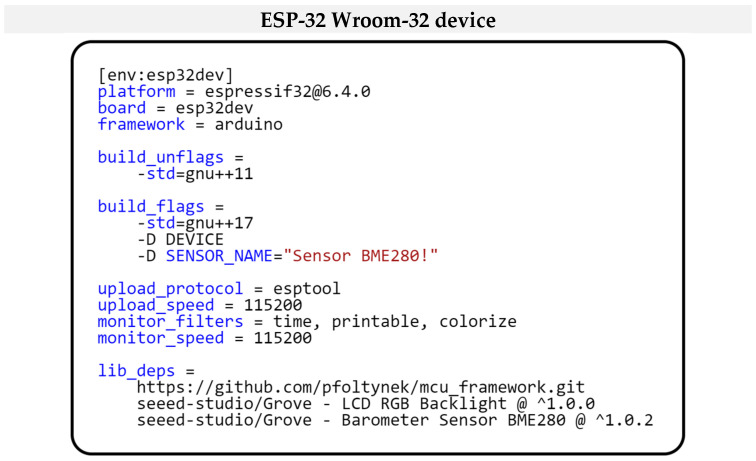
Project configuration for ESP32 Wroom-32 Microcontroller.

When comparing the current configuration of our project, as shown in Figure 24, to the project configuration used in the first example for the “ESP32-WROOM-32” microcontroller, several differences can be identified. The first is the absence of DHTPIN switches in the current configuration. This change is because the BME280 sensor uses an I2C interface for communication, eliminating the need to specify a specific GPIO pin. Another difference is in the SENSOR_NAME switch, which is set to the corresponding name of the currently used sensor BME280 for obtaining air temperature and humidity values. Another difference is in the definition of dependent libraries. While in the first example, the project’s dependencies were limited to our modular framework and specific libraries for the hardware peripherals originally used, in this case, the libraries are modified to support newly integrated hardware components such as the BME280 sensor and 16 × 2 LCD display and still use our modular framework library.

As can be seen from our example, changing the hardware peripherals within our framework causes only platform-dependent changes that are already accounted for in the framework’s structure. Figure 23 graphically represents these changes. This example demonstrates that replacing one type of sensor with another does not require any changes in the application logic implemented within individual modules. This flexibility is particularly beneficial in cases where there is a need to increase the accuracy of sensor measurements or to respond to market fluctuations, as seen during the sensor shortage during the COVID-19 pandemic. Our framework is characterized by its flexibility to quickly respond to changes without the need for extensive modifications to applications. This is possible thanks to the modular architecture, which allows for the simple addition, removal, or replacement of components without disrupting the overall functioning of the application. Another advantage is that this approach enables faster development and testing [17,18] of different configurations, which is crucial to innovation and adaptability in a rapidly changing technology environment. This makes our framework an ideal solution for applications requiring high flexibility and scalability. The source code is available on the GitHub portal [16].

### 2.4. Sample Implementation of the Application Using the Modular Framework

**Figure 25:**
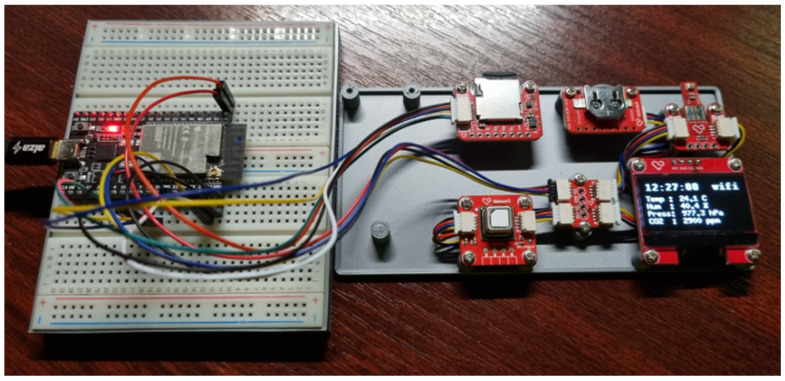
Implementation of advanced task with the modular framework on ESP32-WROOM-32 microcontroller.

In this example, we will present an application that uses our modular framework to integrate different types of sensors and communication modules. The main task of the application is to collect data on temperature, humidity, and air pressure via the BME280 sensor and monitor CO_2_ concentration via the SCD41 sensor. These data are then displayed on a 128 × 64 1.3” OLED display while being sent to the ThingSpeak cloud service [19]. However, we can, of course, also use displays from previous scenarios by appropriately modifying the Sensor–View design pattern of the given *DisplayModule*. In case of using the previous DHT11 module instead of the BME280 module, we will perform the same modification process in the Sensor–View design pattern for the corresponding *TempHumiModule* module. The application also uses the RTC module DS3231, which provides the current time displayed on the OLED display. An SD card module configures the Wi-Fi connection and stores access data to the ThingSpeak cloud service. The configuration data are stored in an ini file format on the SD card. Espressif ESP32-WROOM-32 microcontroller is used as the central element of the whole system. The implementation of this example circuit is shown in Figure 25. Communication between the individual modules is via the I2C communication interface, and only the SD card module uses the SPI bus.

In this example, we will focus in detail on the use of a modular framework in application development. We will explore how the entire project can be effectively divided into individual modules, emphasizing each module’s specific responsibilities. We will also focus on how these modules can work together without compromising their autonomy and independence, which is critical to maintaining a high level of modularity and flexibility in the entire system. The result of this approach is an application that is well-structured and organized but also very maintainable and easy to extend.

**Figure 26:**
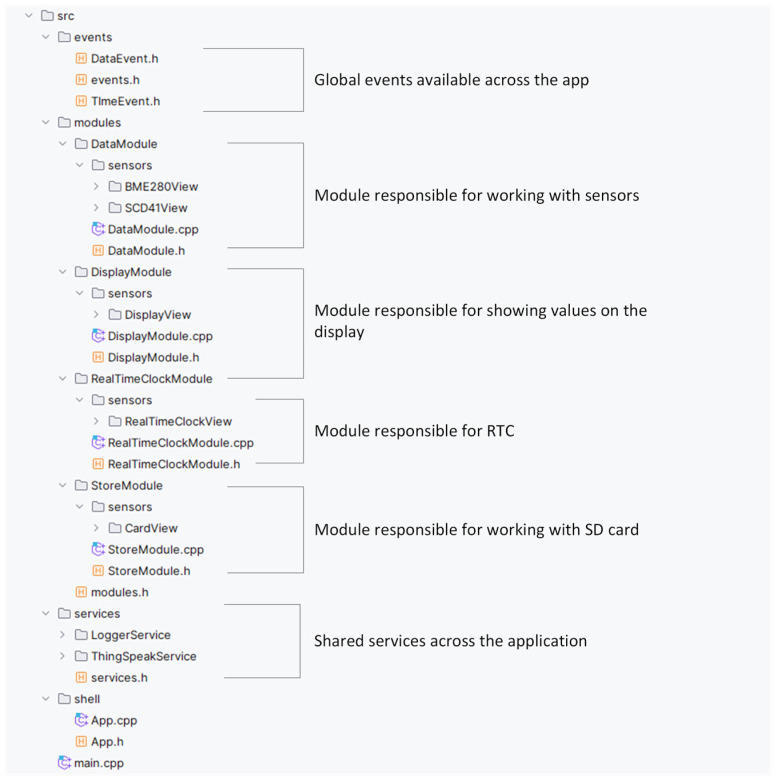
Structure of an advanced modular application for data collection and visualization in a cloud service.

We now look in more detail at the project’s structure, shown in Figure 26. It is essential to pay attention to individual modules. Before introducing each module, we need to notice the global events section, which allows independent communication between modules. Currently, our application uses the following two types of global events:

- *DataEvent*: this event provides the structure of the measured data.
- *TimeEvent*: this event is raised when the time changes.

The section describing individual modules that use these events presents a more detailed view of these events. Another essential part is the Shared Application Services section, where the logging service serves as a universal logger, providing a centralized way of logging across the entire application. There is also the *ThingSpeakService*, which is specifically designed to communicate with the ThingSpeak cloud service.

**Figure 27:**
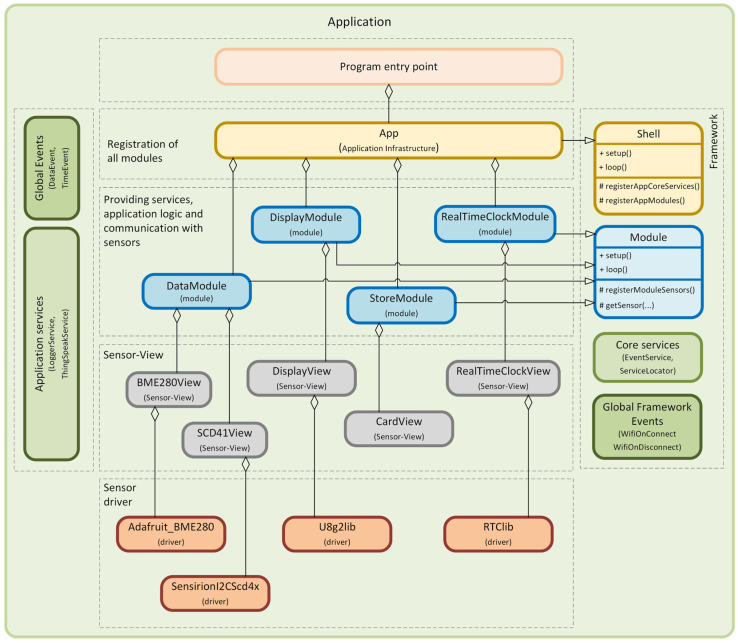
Diagram of component responsibility of our IoT application.

Having explored global events and shared application services, we now move on to a detailed introduction of the individual application modules. We will focus on their specific responsibilities and functions and how they contribute to the application’s overall functionality. This detailed overview will allow us to understand better how the individual components are interconnected and how they work together. Figure 27 illustrates the relationships in our application’s architecture to help developers understand them better. The complete source code for the application is available on the GitHub portal [16].

Now, we will describe the different parts of the application and its responsibilities. The first module we will focus on is the *DataModule*. Its primary role is to collect data from the BME280 and SCD41 sensors and then to provide this information to other parts of the application via the global *DataEvent*. This module is implemented within the *DataModule* class.

As the modular framework prescribes, each module contains a *setup()* method in which it is initialized. Within this module, a 5 s delay is implemented to ensure proper initialization and calibration of the sensors. Another important method prescribed by the framework is the *registerModuleSensors()* method. In this method, individual sensors are registered and implemented using the Sensor–View design pattern, see Figure 27. Specific data structures are created for communication with the module, transferring data from the sensor to the module. This approach is chosen to increase the generality and minimize the dependency of the module’s application logic on the platform part that uses drivers for specific sensors. This approach allows for a high degree of modularity and separation of application and hardware aspects. The SCD41 sensor uses the SCD41Data structure to provide values of temperature, humidity, and concentration of CO_2_ particles, and the BME280 sensor uses the BME280Data structure to provide values of temperature, humidity, and air pressure.

The last method prescribed by the framework is the *loop()* method. Within this method, it is important to highlight the mechanism where the *loopProcessing()* method is called to retrieve data from the sensors every 5 s. The time-based approach is chosen to allow individual modules to respond to different time intervals independently of other modules. This ensures the flexibility and autonomy of each module in the system. As previously mentioned, the *loopProcessing()* method retrieves data from the registered BME280 and SCD41 sensors. Finally, these data are published as a global event through the EventService class, which signals to other parts of the application that new measured values are available. The class for this global event is designed as an immutable object to ensure data consistency and security when shared between different application parts.

We have now described in more detail one of the two modules that provide the data for the display. We will now move on to describe the second module, represented by the *RealTimeClockModule* class. This module obtains time values through communication with the DS3231 RTC module. As described in the previous module, each module implements several methods that are prescribed by the framework. In the *RealTimeClockModule* class, the *setup()* method initializes the RTC of the DS3231 module. This module is integrated into the system using the *registerModuleSensors()* method, where it is registered. The Sensor–View design pattern is used to communicate the module with the sensor, which contributes to the separation of the application logic of the module from the platform-dependent parts of the sensor. This approach allows the module to communicate with the RTC module without being directly affected by the specifications or limitations of the specific hardware. An important aspect of this implementation is using data structures to minimize the module’s dependency on the hardware part.

Another method within the *RealTimeClockModule* class is the *loop()* method. In the *loop()* method, the *loopProcessing()* method is called periodically, every 0.5 s, to get the time component from the RTC module. We chose this interval to allow the display time to be updated every second. During the processing of the *loop()* method, the registered sensor is accessed, and the current time is obtained. Subsequently, this time is published via a global *TimeEvent* to the entire application. These events are also implemented as immutable objects to ensure data consistency and security when sharing data between different application parts.

Now that we have discussed the *DataModule* and *RealTimeClockModule*, which provide data at specific time intervals, we will move on to a description of the *DisplayModule*, which displays this data on the screen. Again, we will first describe this module’s *setup()* method. At the beginning of the *setup()* method, the hardware display module is first registered in the *registerModuleSensors()* method. This behavior is provided because the *DisplayModule* inherits from the *Model* class, which is implemented in the framework. This dependency can be seen in Figure 27. In the next part of the *setup()* method, individual global events are gradually subscribed through the *EventService* class. The first global registered event is *TimeEvent*, provided by the *RealTimeClockModule*. The processing of this subscribed event is performed using the *handle_TimeEvent(…)* method, and the complete code of this module is in [15]. In this method, the hardware display module, implemented using the Sensor–View design pattern, is accessed, and a method is called to provide a redraw of the time displayed on the display. Another subscribed global event is the *DataEvent*, which the *DataModule* provides. This module provides us with the measured values from the BME280 and SCD41 sensors. This event is processed within the *handle_DataEvent(…)* method [15]. Subsequently, a method used on this hardware module that displays the measured values on the display. Other global events for which we register subscriptions are *WifiOnConnect* and *WifiOnDisconnect*. These events are part of our modular framework and provide information about the connection or disconnection of a given microcontroller from the Wi-Fi network. To handle these global events, the methods (*handle_WifiOnConnect(…)*, *handle_WifiOnDisconnect(…)* [16]) are called to react to changes in the connection status of the microcontrollers to a given wireless network. Within these methods, the hardware display module is accessed by default to display the status of the connection to the Wi-Fi network. Another method implemented in the *DisplayModule* is the *loop()* method, which does not implement any application logic. *DisplayModule* responds to global events provided by other modules, and therefore, no regular activity is required within this method.

The last module we will introduce in this example is the *StoreModule*. Its task is to obtain the settings through the SD card module and then initialize the shared *ThingSpeakService*, which provides communication with the ThingSpeak cloud storage over a Wi-Fi network. We now introduce the implementation of the *StoreModule* class. Traditionally, we start by describing the *setup()* method. This method registers the SD card hardware module using the Sensor–View design pattern. This registration is performed using the *registerModuleSensors()* method. The SD card hardware module is then accessed using the *getSensor()* method. The *Module* class of our framework prescribes all the mentioned methods. After accessing the SD card’s hardware module, we receive settings for connecting to the Wi-Fi network and logging into the ThingSpeak cloud storage. Retrieving these settings is essential for configuring the network connection and communicating with the cloud. The next step of the *setup()* method will initialize the *ThingSpeakService*. This initialization is based on the settings stored on the SD card. Another implemented method in the *StoreModule* is the *loop()* method. Since the configuration is loaded when the application is initialized, i.e., when the microcontroller starts, implementing anything within the *loop()* method is unnecessary.

**Figure 28:**
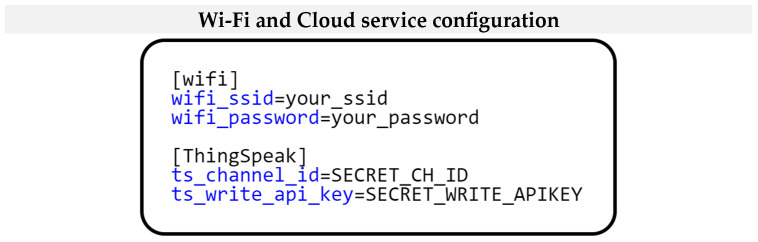
Project configuration for Wireless and cloud communication.

Figure 28 shows the format of the *ini* configuration file stored on the SD card. As we can see, the *ini* configuration file has two sections. The first section is Wi-Fi, which contains the settings for connecting to the Wi-Fi network. The second section is ThingSpeak, where the settings for connecting to the ThingSpeak cloud service are stored. This configuration format allows users to customize the application’s network settings according to their specific needs and requirements.

**Figure 29:**
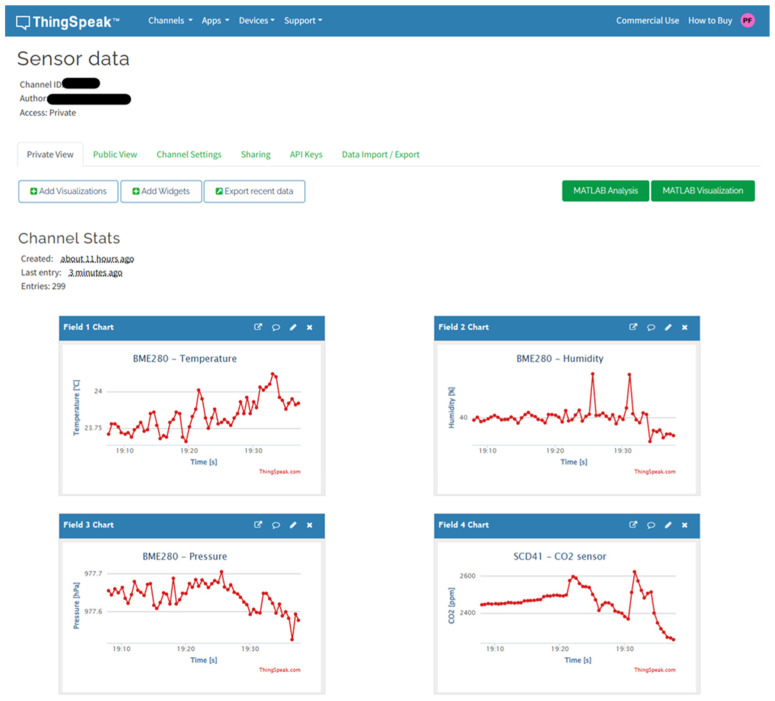
Measured data visualized in the ThingSpeak cloud service.

The initialization of the shared *ThingSpeakService* is provided in the *setup()* method of the *StoreModule*. This service is registered as a global application service via the *ServiceLocator* class, which is part of our framework and offers the *init(…)* method for its initialization. In its constructor, the *ThingSpeakService* subscribes to the global *DataEvent* provided by the *DataModule*. The *EventService* class is used to subscribe to the given event. The constructor is followed by the implementation of the *init(…)* method, where the input arguments correspond to the configuration for the Wi-Fi network and the connection to the ThingSpeak cloud platform. Within the *init(…)* method, the private *wifiConnectStatus()* method is then called, which contains the algorithm responsible for connecting to the Wi-Fi network. Figure 29 shows the ThingSpeak cloud service portal where the data from our example are visualized.

It is important to emphasize that shared services implemented using our framework are considered platform-dependent parts. The platform dependency of these services should generally be based on implementations dependent on the development platform, such as libraries for Wi-Fi network access or libraries for communicating with cloud storage. Hardware modules should not be accessed directly within shared services. Framework modules such as *DataModule*, *RealTimeClockModule*, *DisplayModule*, or *StoreModule* are used for this purpose. Their architectural design is adapted to separate hardware dependencies from the rest of the application. The implemented example shows that specific patterns are often repeated when describing modules. This is because the architecture of the modules is designed to give programmers clear guidance on how to develop the application. This makes the code easier to maintain and increases its readability. This architectural concept makes it possible to modify and extend the application without having to change the application logic.

**Figure 30:**
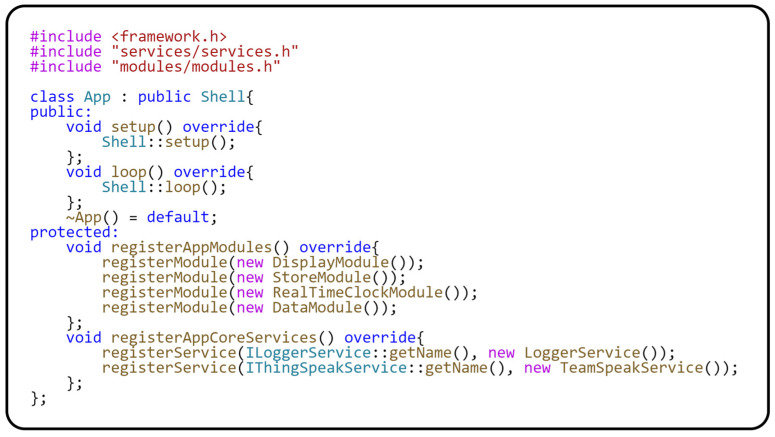
Registration of the module and shared services in the application.

We now describe the implementation of the *App* class, which is responsible for general module management. This functionality is provided by the fact that the *App* class inherits from the *Shell* class, which is part of our modular framework. Furthermore, the *App* class is fully shielded from any hardware dependencies, making it platform-independent. The following code in Figure 30 shows us the implementation of the *App* class. The structure of the *App* class is designed to manage a modular application clearly and efficiently. The main parts of the *App* class are divided into the *registerAppCoreServices()* method, which registers the core services used across the application. In our case, two services are registered here, *LoggerService* and *ThingSpeakService*. The *LoggerService* is responsible for a centralized logging mechanism that systematically monitors and logs significant events in the application. This service is essential for efficient debugging and monitoring of application behavior.

The second service is *ThingSpeakService*, which is responsible for sending the measured data to the ThingSpeak cloud service. This service is key in connecting our application to the cloud environment, enabling remote data collection and analysis. Another critical method in the *App* class is the *registerAppModules()* method, which allows us to register all the modules used in the application. It is important to note that the order in which the modules are registered significantly impacts the application’s overall behavior, as the *setup()* and *loop()* methods of each module are called in that order. The application entry point depends on the development platform and provides initialization and execution of the entire application.

**Figure 31:**
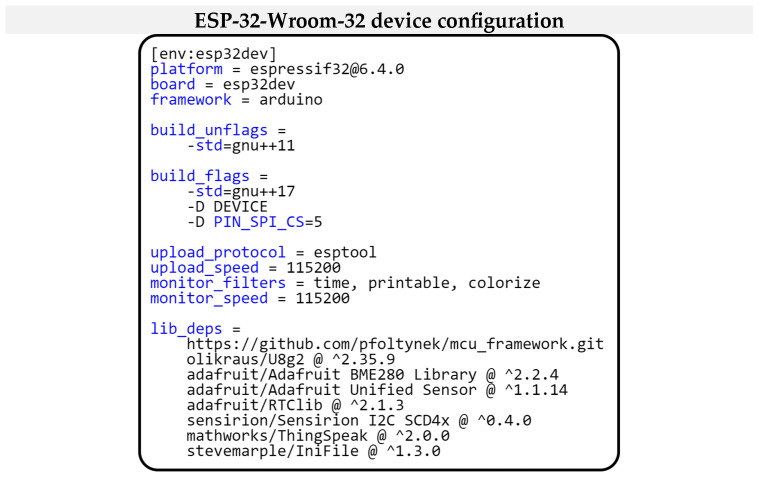
Project configuration for measurement and data visualization in the cloud service.

We will now look into the configuration of the project in more detail. Figure 31 shows the configuration for this project. The configuration indicates that the drivers specified in the *lib_deps* section are used to communicate with the hardware modules. In addition to the drivers for the hardware modules, there is also a library for the communication interface to the ThingSpeak cloud service, explicitly represented by the *mathworks/ThingSpeak* library. Furthermore, the *stevemarple/IniFile* library, which is responsible for processing *ini* files, is listed here. The library that implements our modular framework is represented in the project via a link to GitHub, where its complete implementation is located.

In our example, we are communicating with the ThingSpeak service. Still, if we need to switch to another cloud platform, e.g., Azure [20], we must create a new core service, which we register only in the application context. Within the service, only the subscription of the data-providing event will change.

In this example, we have shown how an application’s architecture can be structured into individual modules with clearly defined responsibilities. We did not focus on a detailed presentation of the implementation of the platform-dependent parts since, from the point of view of the application logic, these parts are straightforward. Their implementation is comparable to the creation of smaller applications specifically designed for individual sensors. We have also shown how individual modules can communicate effectively with each other. We have created platform-shared services that do not directly access hardware elements, but use a platform-dependent part within the development environment. This architectural design makes the application scalable and sustainable in the long term. This application design approach provides programmers with a clear framework for implementing application logic, supporting efficient development and adaptability to changing requirements and technology trends within the rapidly evolving Internet of Things (IoT) industry [21].

## 3. Discussion

During our research on creating software support for the increasingly rapidly growing segment of microcontrollers and the subsequent design of an architectural approach to software development, it is important to answer fundamental questions about the modular framework. We answer these questions in this section.

- In what cases is it appropriate to use a modular framework?

A modular framework is good to use for applications where long-term maintenance and extensibility of the application are planned. At the same time, if to develop an application with many different sensor variants, it is also a good idea to choose this modular framework for developing an application on an embedded system [22]. If it is a large project on which several programmers or teams with different expertise work, then it is possible to distribute the development of such an application to individual teams (programmers) that develop specific modules, and then the application is built.

- Is it always advantageous to use a modular framework?

The modular framework is not always good for developing any application on an embedded system. If it is a single-purpose application where no further development is expected, it is better to use existing approaches for application development, such as procedural or OOP approaches. This is because the introduction of a framework brings some time overhead and code complexity, which pays off more for long-maintainable applications than for single-purpose applications.

- What requirements does the framework place on developers, and what does it require for hardware modules?

We will not use the modular framework on 8-bit chips for controlling and managing simple applications, but it is advantageous, especially for applications where high variability, testability, and maintainability are expected [23]. The framework is suitable for different types of developers. Those who write the application logic of the module may not know how to access the different sensors or what the different communication protocols are to communicate with them. They may be removed from these aspects because of the Sensor–View design pattern.

Another type of programmer is the low-level programmer, for communicating with individual sensors. The latter, in turn, may not know anything about the modular framework and how communication between modules occurs. Hence, the framework is ideal for teams that are focused on efficiency in a particular area of development. Not all team members need to know the architectural approaches, but the senior or team leader should know and cover this.

- What does a modular framework bring in terms of application testability?

Testability is made more efficient by the modular design and structure of the application. The application logic is divided into individual modules, and during the development and subsequent modification of the application, it is not expected to intervene in all parts of the application at once but only in specific modules. Therefore, when retesting, the modules that have not been modified are not checked. Another great advantage and efficiency improvement of application testing is the implementation of Solid principles and programming towards interfaces where we can effectively test HW resources without the presence of hardware. This feature allows us to automate testing during any modification, and final testing on hardware is only performed when the application is released to production.

- What is the essence of the modular framework?

The essence is to write applications that are maintainable and extensible. This is ensured by the basic building block of the framework, the module. To ensure that the application logic of the module is platform-independent from the hardware part, we need to use a new design pattern called Sensor–View, which provides us with an abstract layer to work with the data provided by the sensor.

- Is this framework more demanding than a procedural or OOP approach?

The following Table 1 summarizes the complexity, advantages, and disadvantages of all three principles of microcontroller application development.

- Is it possible to perform a comparative analysis of the framework with development frameworks?

Development frameworks can be divided into general frameworks for a wider range of microcontrollers and frameworks for specific types of microcontrollers. Arduino, Zephyr, FreeRTOS introduced the first ones, while AVR, ESP–IDF, MBED, STM32Cube, and others represent the second group. It is important to say that these development frameworks provide tools, libraries, and structures for creating software applications.

In contrast, our universal modular framework is an application framework. An application framework is designed to facilitate the rapid build of applications with a higher level of abstraction. It focuses on providing a structure for a specific type of application. We want to highlight the fact that our application framework can be developed in different development frameworks. Our modular FW is an application framework that is fully implemented in an embedded environment with SOLID principles and design patterns for working with microcontrollers. Table 2 shows the differences between the frameworks.

- How was our new design pattern created?

Our new *Sensor–View* Design Pattern is inspired by the MVC (*Model-View-Controller*) architecture [24,25]. It is structured to separate the platform-dependent sensor controllers (*Sensor*) from the platform-independent interface for interacting with these sensors (*View*). This model allows easy integration and replacement of sensors without the need to modify the rest of the application.

*Sensor:* This layer is platform-dependent, and each sensor driver is specific to the type of hardware or microcontroller. It handles low-level communication with the hardware sensors, processes data from the sensors, and provides these data in a form that can be further processed by the application logic.

*View:* Because it is platform-independent, this layer provides a consistent interface for the application to interact with sensors independent of the specific hardware. The View may include logic to customize or transform sensor data to make it suitable for use in the application. Because View is decoupled from particular sensor drivers, sensors can be easily replaced or updated without changing application logic.

- How does a modular framework contribute to solving current shortcomings in application development?

Our modular framework eliminates critical shortcomings associated with application extensibility and testability. These shortcomings, especially common in procedural programming, are eliminated by using SOLID principles and implementing proven design patterns:

**Improved testability:** The framework uses the Single Responsibility (S) principle from SOLID approaches, meaning each part of the code has a clearly defined responsibility. This means that when making changes, testing the entire application is unnecessary, but only the part where the change occurred. Testing is further simplified by implementing the Dependency Inversion (D) principle through the Service Locator. This technique allows for effectively mocking hardware and platform-specific components, greatly facilitating development and testing.

**Application Extensibility:** In procedural programming, changes to function parameters or adding new functionalities can often lead to chain modifications across the entire application. Our framework eliminates this problem through a modular approach, where individual modules are designed as loosely coupled. This means that each module is fully independent and can be run independently without dependence on the others. The framework implements the Event–Service service to support loose coupling between modules, which uses a variant of the Publish–Subscribe design pattern. This approach allows a person to effectively extend the application’s functionality by adding new modules without interfering with existing code. This ensures high flexibility and easy maintainability.

By implementing SOLID principles and specific design patterns (a detailed description of the implementation is in the article [9]), our framework ensures effective solutions to problems associated with the traditional procedural approach, facilitating application development, expansion, and long-term maintenance.

## 4. Conclusions

In our article, we have presented detailed application scenarios that illustrate the capabilities and limitations of our modular framework. These application scenarios tested its robustness. A key finding was that our framework is designed to be independent of a specific development environment, be it Arduino or ESP–IDF, and simultaneously flexible to different types of microcontrollers. This independence and flexibility allow us to quickly respond to technological changes and adapt applications to different sensors without changing the basic application logic. Because of how our framework is designed, we can separate the platform-specific parts from the platform-independent parts, which is crucial for the long-term sustainability, testability, and extensibility of applications. This approach allows developers to quickly adapt applications to different usage scenarios and user needs without completely redesigning or reworking existing code. In addition, we demonstrated how modules can effectively communicate with each other and how shared services can be created that are independent of hardware components but use platform-dependent parts. This approach reduces complexity and increases the clarity of the application’s overall design. As a result, our framework supports developers in creating robust, adaptable, and scalable applications. It emphasizes a modular and clean design that increases development efficiency and enables easy deployment of new functions or integration of new technologies. In the last example, we also explored some advanced concepts and architectural designs that are applicable in the broader context of application development. These concepts offer helpful guidance for building applications that are ready for future challenges and the changing technology landscape, especially in the IoT field. In this way, our examples serve not only as a demonstration of the possibilities of our framework but also as inspiration for future innovative projects.

## Figures and Tables

**Table 1:** Pros and cons of three principles of microcontroller application development.

Procedural Programming	
**Pros**	- easy to implement - lower resource requirements - code is straightforward - fast coding of a given task
**Cons**	- poor extensibility of the application - impossibility of testing - modifying a part of the code causes a chained modification of the whole application - rewriting the application is easier than implementing the modification - application logic is strongly coupled
**OOP**	
**Pros**	- we can develop applications with the support of more efficient language constructs that take advantage of the OOP concept - OOP allows us to write the application better for long-term maintenance, increases the readability and reusability of the code - OOP clearly defines the approach of how to work with data and who accesses the data under what conditions
**Cons**	- the application using OOP, although it is well structured and fully exploits the possibilities of language construction of the program, does not define how to write the application for long-term maintenance, extensibility, and testability - the application logic is encapsulated in individual objects, but these objects are still strongly linked within the program
**Modular Framework with Design Patterns**	
**Pros**	- an application consists of loosely coupled modules that encapsulate the application logic - the application can be better tested and then extended and maintained - the application logic is implemented within the module, and communication with sensors is platform-independent - it is possible to develop and test the application without the presence of hardware
**Cons**	- using the Modular Framework is not ideal for deploying trivial single-purpose applications because engaging the Modular Framework requires some overhead for project configuration and maintenance - it puts demands on the programmer, who must understand the concepts and change his or her thinking from procedural programming to a more abstract level - in certain cases, higher memory requirements are possible

**Table 2:** Comparison of development and application frameworks.

Feature	Development Framework	Application Framework
**Level of Abstraction**	Low (close to hardware)	High (close to application)
**Target Usage**	For developers working at a low level (hardware integration, drivers)	For developers creating high-level application
**Focus**	Hardware interaction, peripherals	Rapid application development
**Framework Examples**	Arduino, ESP–IDF, Zephyr, STM32Cube, MBED, FreeRTOS	Our Universal Modular FW

## Data Availability

The data and sources codes presented in this article are available on request from the corresponding author.
